# HJURP indicates poor prognosis of female breast cancer by promoting cell proliferation and migration

**DOI:** 10.1016/j.gendis.2023.101176

**Published:** 2023-11-23

**Authors:** Wenquan Chen, Hao Li, Dandan Wang, Sujin Yang, Junchen Hou, Honglei Zhou, Jinhai Tang, Jian Zhang

**Affiliations:** a Department of General Surgery, The First Affiliated Hospital with Nanjing Medical University, Nanjing, Jiangsu 210029, China; b Department of General Surgery, Nanjing Gaochun People's Hospital, Nanjing, Jiangsu 211300, China

Female breast cancer (BCa) has overtaken lung cancer as the most diagnosed cancer worldwide.^1^ The overall survival for BCa patients without metastasis for five years is over 80%.^2^ However, relapse and metastasis remain the primary challenge for BCa. In various carcinomas, Holliday junction recognition protein (HJURP) has shown up-regulated patterns and belongs to the CENP-A pre-nucleosomal complex.^3^, ^4^, ^5^ HJURP's expression pattern, roles, and prognosis remain largely unknown in BCa. Nevertheless, we investigated HJURP's expression in BCa and explored its possible role in cancer development. We found that HJURP was significantly higher in BCa tissues and associated with pathological characteristics, and down-regulation of HJURP in BCa cells could suppress the proliferation and migration of MDA-MB-231 cells through the PI3K-AKT pathway. A significant role for HJURP in BCa progression has been identified, and it could provide a valuable biomarker for the diagnosis and prognosis of BCa. Also, considering HJURP as the novel diagnosis and treatment target will provide new strategies for early diagnosis, precise treatment, and effective prevention of BCa.

The first objective was to determine the expression profile of HJURP in BCa and its relation to BCa features. A TCGA analysis found a significantly higher expression of HJURP in BCa tissues and the highest expression in triple-negative breast cancer (TNBC) (Fig. 1A, B). HJURP expression was negatively associated with the estrogen receptor and progesterone receptor, but positively associated with human epidermal growth factor receptor-2 (Fig. 1C–E). In addition, HJURP expression was significantly higher in TNBC or basal-like subtype (Fig. S1A–C); HJURP was increased in patients with BRCA1/2 gene mutations or lymph node invasion and high-stage pathology (Fig. S1D–I). These findings suggest that HJURP may be associated with tumor malignancy in BCa.

**Figure 1:**
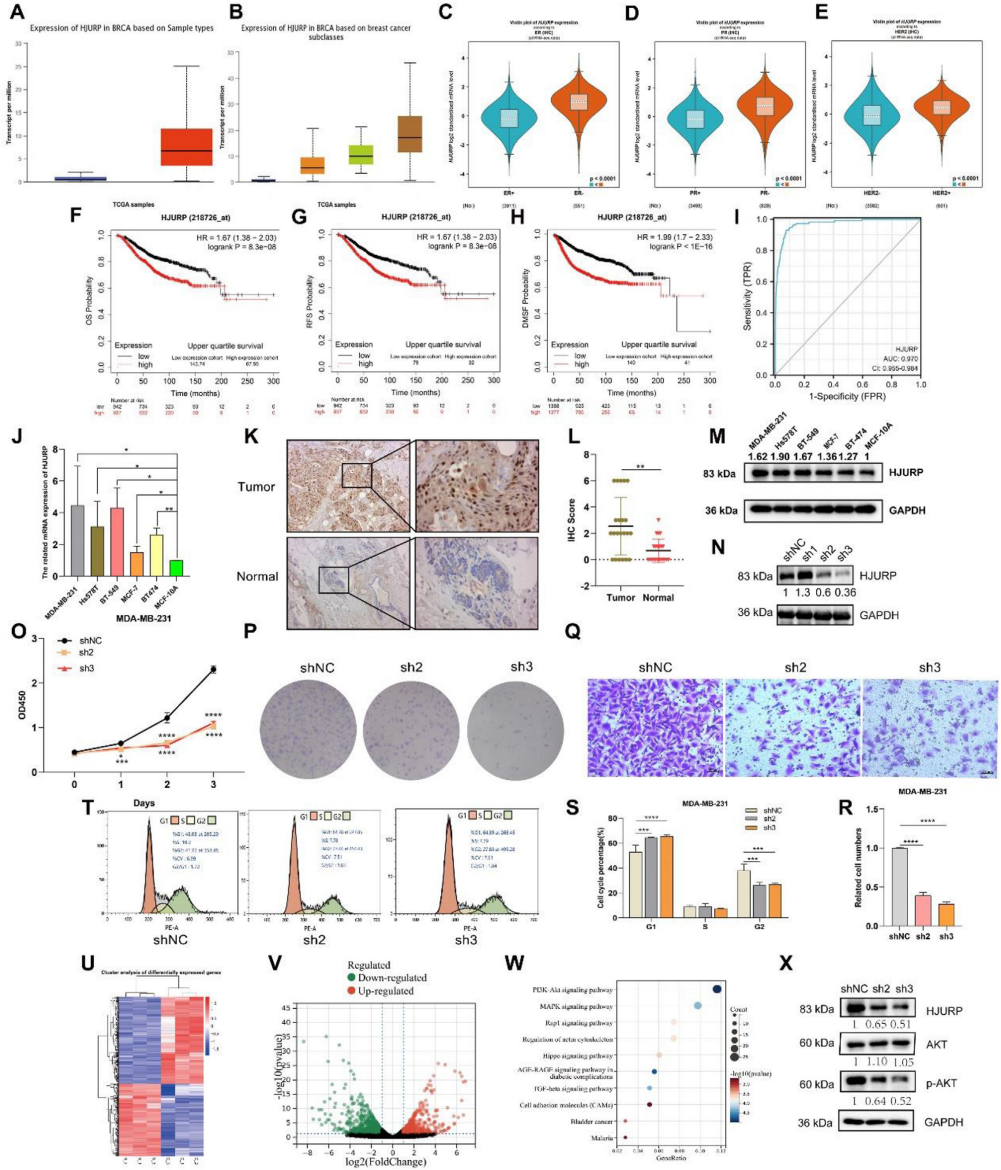
Identification the expression and roles of HJURP in BCa. Histogram of HJURP expression in **(A)** primer BCa and normal tissues, **(B)** BCa major subtypes. HJURP profiles in BCa with different molecular status **(C)** ER, **(D)** PR, **(E)** HER2. The association between HJURP and BCa patients' survival **(F)** OS, **(G)** RFS and **(H)** DFS. **(I)** Sensitivity and specificity of HJURP as a BCa diagnostic marker. **(J)** mRNA expression of HJURP in BCa cells and MCF-10A cell by RT-qPCR. **(K)** HJURP expression in BCa and adjacent tissues by IHC (magnification, × 100, scale bar 100 μm, magnification, × 400, scale bar 50 μm) and **(L)** each sample score. **(M)** Protein of HJURP in BCa cells and MCF-10A cell. **(N)** Knocking down HJURP in MDA-MB-231 cells by shRNA. **(O)** CCK-8 assay of MED-MB-231 cell. **(P)** Clone formation assay of MED-MB-231 cell. **(Q**, **R)** Transwell assay of MED-MB-231 cell (magnification, × 200, scale bar 50 μm). **(T,S)** Cell cycle detection in two groups by flow cytometry. ^∗^*P* < 0.05, ^∗∗^*P* < 0.01, ^∗∗∗^*P* < 0.001, ^∗∗∗∗^*P* < 0.0001.

Analysis conducted above indicated that HJURP expression in BCa tissues increases with tumor malignancy. However, it is still unclear whether HJURP expression is related to BCa survival. To explore this relationship, we analyzed data from TCGA through the Kaplan–Meier plotter, which revealed that the high HJURP expression was associated with poor overall survival (Fig. 1F), relapse-free survival (Fig. 1G), and distant metastasis-free survival (Fig. 1H). In addition, we examined HJURP's relationship with survival in BCa patients with different characteristics. A negative association was observed between HJURP expression and overall survival in patients with estrogen receptor-positive or human epidermal growth factor receptor-2-positive BCa, but no significant difference was found in TNBC patients (Fig. S1J–L). Although patients with high HJURP expression had worse overall survival than the lower expression group in lymph node metastasis BCa patients, no statistical difference was observed (Fig. S1M). However, in non-lymph node metastasis patients, high HJURP expression was associated with poor overall survival (Fig. S1N). Furthermore, we comprehensively analyzed the survival of patients with lymph node metastases and other characteristics. HJURP expression was associated with shorter overall survival in lymph node metastasis plus estrogen receptor-positive or human epidermal growth factor receptor-2-positive BCa group, but not with TNBC (Fig. S1O–Q), which might be due to the limited number of patients in this group. Based on its expression and prognosis patterns, HJURP may play a critical role in BCa. However, the diagnostic value of HJURP remains unclear. We found that HJURP showed outstanding specificity and sensitivity for BCa diagnosis, with an area under the curve of 0.97 (Fig. 1I). Therefore, HJURP has great potential in the diagnosis of BCa.

When investigating the impact of HJURP on immunity, we discovered that HJURP participated in chemokine genes, chemokine receptors, major histocompatibility complex genes, and immune-inhibitory and immune-stimulatory gene regulation (Fig. S1R). Association analysis between HJURP and immune cells indicated that HJURP was generally associated with macrophages and had a positive correlation with M1 macrophages, but a converse correlation with M2 macrophages. Additionally, our results suggested that HJURP might closely influence the biofunction of CD4^+^ TH2 T cells (Fig. S1S). Finally, we investigated the potential connection between HJURP and immune checkpoint. Our analysis revealed that HJURP was associated with a series of checkpoints, such as CD40, CD70, CD80, CD274, CTLA4, ICOS, and TNFRSF8/9 (Fig. S1T).

The expression of HJURP mRNA in various BCa cell lines was detected by RT-qPCR. HJURP was observed highly expressed in BCa cell lines (Fig. 1J). The immunohistochemical assay showed that the level of HJURP protein was significantly higher in tumor tissues than in normal mammary gland tissues (Fig. 1K, L). In BCa cells, HJURP protein was also significantly increased (Fig. 1M). To further investigate the function of HJURP, we successfully knocked down its expression in MDA-MB-231 BCa cells using shRNAs (Fig. 1N). We selected shRNA2 and shRNA3 as candidates for subsequent experiments. CCK-8 and colony formation assays demonstrated that the proliferation ability of MDA-MB-231 cells was significantly reduced when HJURP was down-regulated (Fig. 1O, P). Furthermore, the transwell assay showed that the invasive ability of MDA-MB-231 cells was weakened in the HJURP knockdown group compared with the control group (Fig. 1Q and R). Since HJURP is necessary for the process by which CENP-A specifically targets centromeres to promote cell mitosis, we analyzed the role of HJURP in cell cycle progression using flow cytometry. Knockdown of HJURP led to an increase in the number of cells arrested in the G1 phase, while the number of cells in the G2/M phase decreased (Fig. 1S, T).

Finally, we performed RNA-seq analysis in HJURP-knockdown MDA-MB-231 cells and control groups. The analysis identified 269 down-regulated genes and 312 up-regulated genes in the HJURP knockdown group compared with the control group (Fig. 1U, V). KEGG pathway analysis showed that the differentially expressed genes were enriched in cancer-related pathways such as the PI3K-AKT signaling pathway, MAPK signaling pathway, Rap1 signaling pathway, Hippo signaling pathway, and TGF-beta signaling pathway (Fig. 1W). In MDA-MB-231 cells, the knockdown of HJURP resulted in a significant decrease in p-AKT expression with no change in the total amount of AKT (Fig. 1X). Enrichment analysis of the differentially expressed genes suggested that HJURP might regulate breast cancer progression through the PI3K-AKT signaling pathway.

Our bioinformatics analysis and cell biology experiments revealed that HJURP is abnormally overexpressed in breast cancer and is associated with a poor prognosis. Knocking down HJURP successfully inhibited the malignant biological behavior of BCa cells. Ultimately, we believe HJURP can be a promising marker for BCa diagnosis and is a potential treatment target.

## Ethics declaration

The study was conducted in accordance with the Declaration of Helsinki, and approved by the Ethics Committee of Jiangsu Province Hospital (No. 2020-SR-477).

## Author contributions

The concept and design were carried out by JHT and JZ. Data acquisition was performed by HL and DDW. Data was analyzed by SJY and JCH. Experimental studies and manuscript editing were executed by WQC. The manuscript was revised by HLZ. All authors approved the submission of the final version of the manuscript to *Genes & Diseases*.

## Funding

This research was funded by the National Natural Science Youth Fund of China (No. 82103626) and the Nanjing Gaochun People's Hospital Institute-Level High-Quality Scientific Research Projects (China) (No. GYK-2022-002).

## Conflict of interests

The authors declare no conflict of interests.
