# Supplementary material for: HJURP indicates poor prognosis of female breast cancer by promoting cell proliferation and migration
Source: Genes Dis. 2023 Nov 23;11(6):101176. doi: 10.1016/j.gendis.2023.101176 (PMC11320451; doi:10.1016/j.gendis.2023.101176)
Supplement: Multimedia component 1 [file mmc1.docx]

**Supplementary Material**

**Methods and Material**

**Ualcan, Timer2 and Sangerbox**

UALCAN (http://ualcan.path.uab.edu/), a comprehensive and user-friendly web resource for analyzing cancer OMICS data based on The Cancer Genome Atlas (TCGA) database was used to explore the expression of HJURP in breast cancer (BCa) and Timer2 (http://timer.cistrome.org/), Sangerbox (http://sangerbox.com/Index) were used to analyze the relationship between HJURP and immunity

**Breast Cancer Gene-Expression Miner**

We utilized Breast Cancer Gene-Expression Miner v4.8 (http://bcgenex.ico.unicancer.fr/BC-GEM/GEM-Accueil.php?js=1) to study the relationship between HJURP expression profiles and various BCa characteristics, such as estrogen receptor (ER), progestogen receptor (PR), human epidermal growth factor receptor 2 (HER2), nodal status, SBR, NPI, triple-negative status, basal-like status, PAM50 subtypes, pathological tumor stage, and BRCA1/2 mutation.

**Kaplan-Meier Plotter**

Kaplan-Meier Plotter (http://kmplot.com/analysis/index.php?p=service) was used to analyze whether the expression of HJURP was associated with the survival of BCa patients with different characteristics, such as ER, PR, HER2, or lymph node status through TCGA database.

**Samples collection**

BCa specimens from the Department of General Surgery at Nanjing Medical University's First Affiliated Hospital in 2022 (supported by 2020-SR-477). Patients or their next of kin provided informed consent, and breast tumor and normal tissues were rapidly frozen using liquid nitrogen. and the IHC score of BCa samples used in this study were shown in Table S1.

**Cells culture and RT-qPCR**

Breast epithelial cell line MCF-10A and human breast cancer cell lines MCF-7, MDA-MB-231, Hs578T, BT-549, and BT-474 were obtained from the Cell Bank of the Chinese Academy of Sciences. The MCF-7, MDA-MB-231, Hs578T, BT-549, and BT-474 cell lines were cultured in Dulbecco’s modified Eagle’s medium (DMEM) high glucose (KeyGEN, China) or RPMI1640 (KeyGEN, China) medium supplemented with 10% fetal bovine serum (FBS) (Wisent, CAN), 100 μg/ml penicillin, and 100 U/ml streptomycin. The MCF-10A cell line was cultured in MEBM Basal medium (Lonza, Switzerland). All cells were incubated at 37°C with 5% CO2. Total RNA was extracted from the cells using TRizol (Invitrogen, USA). The HiScript II 1st Strand cDNA Synthesis Kit (Vazyme China) was used to reverse transcribe the total mRNA from the cells according to the manufacturer's instructions to synthesize cDNA. RT-qPCR was performed to measure the HJURP mRNA expression levels using the ChamQ SYBR qPCR Master Mix (Vazyme China). The primer sequences designed by PrimerBank are shown in Supplementary Table S2.

**Immunohistochemistry (IHC) and Western blot**

Tissues fixed on glass slides by paraffin embedding were baked at 60°C for 1 to 2 hours. The tissues were then dewaxed in xylene and hydrated with a decreasing gradient of ethanol. After blocking endogenous enzymes and nonspecific binding sites, the slices were incubated overnight at 4°C with HJURP antibody diluted 1:1500 (Proteintech, China). Next, donkey anti-mouse/rabbit secondary antibodies and Streptomyces anti-biotin protein-peroxidase were incubated with the pathological slices for 10 minutes, following the manufacturer's instructions for the IHC Kit (KIT-9710, Maixin, China).

For protein extraction from breast cancer cells, a RIPA lysis buffer (BioteChinaology, China) was used according to the manufacturer's instructions. The protein concentration was estimated using a BCA Protein Assay Kit (BioteChinaology, China). Approximately 15 μg of total protein was separated using 10% SDS-PAGE and transferred onto PVDF membranes (Millipore, USA). The PVDF membranes were blocked for 15 minutes using the QuickBlock™ Western Kit (Beyotime BioteChinaology, China), and then incubated overnight at 4°C with rabbit anti-HJURP (15283-1-AP, Proteintech, China) diluted 1:1000 and mouse anti-GAPDH (Proteintech, China) diluted 1:20000. The corresponding secondary antibodies were incubated with the PVDF membrane for 1 hour at room temperature. Protein bands were visualized using Enhanced Chemiluminescence (ECL) Plus (Yeasen BioteChinaology, China) and imaged with a Bio-Rad ChemDoc XRS (Bio-Rad, USA).

shRNA vectors and HJPR stable knock-down MDA-MB-231 cell

We used a pLKO.1 vector resistant to puromycin to design shRNA vectors that could suppress the expression of HJURP. The sequences of the HJURP shRNA are listed in Supplementary Table S3. To evaluate the knockdown effect of HJURP, we performed a Western blot assay.

**CCK-8, colony formation, cell cycle and transwell assays**

To assess the proliferation activity of BCa cells, we used the Cell Counting Kit-8 (CCK-8) (APExBIO, USA). We cultured 10,000 cells/well in a 96-well plate and measured the OD450 value of cells every 24 hours, four times in total, after counting the cell number. To examine cloning capability, we used 1,500 cells/well for colony formation assays in a 6-well plate. We analyzed the cell cycle of BCa cells stained with PI by flow cytometry (BD Biosciences, NJ) in accordance with the manufacturer's protocols.

For the Transwell assay, we used 24-well plate transwell inserts (Corning, USA). We seeded 2 × 10^4 cells in 200 μL DEME medium without FBS into the upper chamber, while 800 μL of DMEM medium with 20% FBS was added to the bottom chamber. After 20 to 24 hours, we fixed the samples for 30 min using 4% paraformaldehyde and stained all inserts with crystal violet. We observed all pictures using a Zeiss Axio Vert. A1 inverted fluorescent microscope (Carl Zeiss Microscopy GmbH, Germany).

**RNA sequencing assay and data analysis**

To identify downstream genes of HJURP, RNA sequencing was conducted on MDA-MB-231 cells with HJURP knockdown and control groups. Total RNA was extracted using the TRIzol method (Invitrogen, CA, USA) and treated with RNase-free DNase I (Takara, Kusatsu, Japan). A total of 1.5 μg RNA per sample was used to prepare sequencing libraries using the NEBNext® Ultra™ RNA Library Prep Kit for Illumina® (NEB, USA), with index codes added to attribute sequences to each sample. First-strand cDNA was synthesized using random hexamer primer and M-MuLV Reverse Transcriptase (RNase H), followed by second-strand cDNA synthesis using DNA Polymerase I and RNase H. Adenylation of 3’ ends of DNA fragments and ligation with NEBNext Adaptor with hairpin loop structure was performed for hybridization. The samples were treated with USER Enzyme (NEB, USA), and then PCR was performed using Phusion High-Fidelity DNA polymerase, Universal PCR primers, and Index (X) Primer. PCR products were purified using the AMPure XP system, and library quality was assessed on the Agilent Bioanalyzer 2100 system. The libraries were sequenced on an Illumina Novaseq 6000 platform by the Beijing Allwegene Technology Company Limited (Beijing, China) with paired-end 150bp reads generated. Differential expression analysis was performed using the DESeq R package (1.10.1), which provides statistical routines for determining differential expression in digital gene expression data using a model based on the negative binomial distribution. The resulting P-values were adjusted using the Benjamini and Hochberg’s approach for controlling the false discovery rate. Genes with an adjusted P-value < 0.05 found by DESeq were assigned as differentially expressed.

**Figure S1**

**
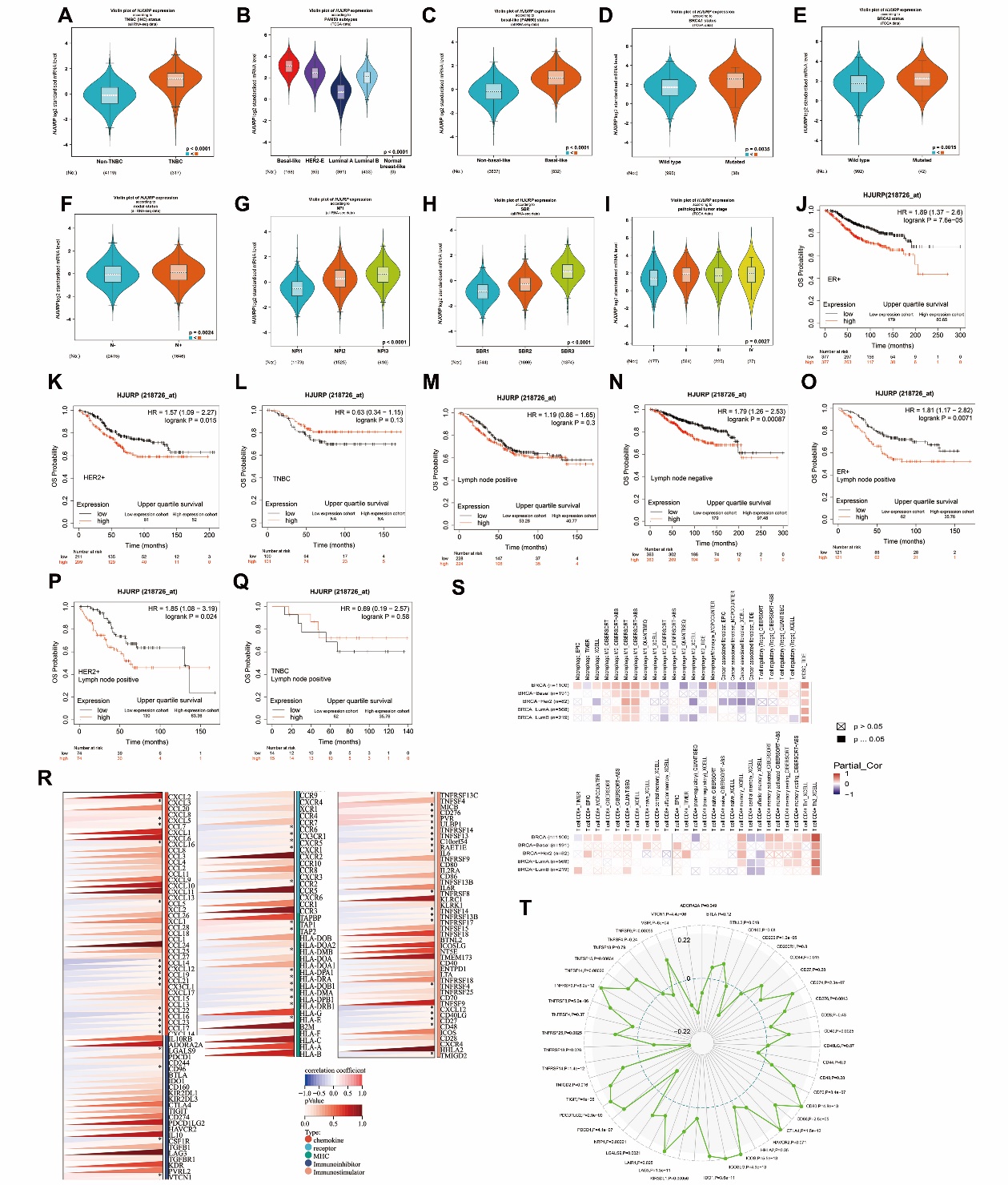
**

**Figure S1:**  Relationships between HJURP and BCa molecular features, prognosis or immunity.

HJURP expression in BCa with different characteristics (**A**) Non-TNBC and TNBC, (**B**) Basal-like, HER2-E, luminal A and Luminal B, (**C**) Non-Basal-like and Basal-like. With different genes mutation (**D**) BRAC1 mutation, (**E**) BRAC2 mutation. With different (**F**) lymph node status, (**G**)NPI status, (**H**)SBR status and (**I**) pathological stages. The association between profiles of HJURP and BCa patients’ survival (**J**) ER+, (**K**) HER2+, (**L**) TNBC, (**M**) lymph node positive, (N) lymph node negative BCa patients. OS in (**O**) ER+ plus lymph node positive, (**P**) HER2+ plus lymph node positive and (**Q**) TNBC plus lymph node positive. (**R**) The association between HJURP and immune regulated genes. (**S**) The relationship between HJURP and immune cells. (**T**) The links between HJURP and immune checkpoints.

**Table S1:** IHC score

| Sample | Tumor tissue | | | Paracancerous tissue | | |
| --- | --- | --- | --- | --- | --- | --- |
|  | Density score | Intensity score | Total  score | Density score | Intensity score | Total  score |
| 1 | 1 | 2 | 2 | 3 | 1 | 3 |
| 2 | 3 | 1 | 3 | 0 | 1 | 0 |
| 3 | 0 | 2 | 0 | 0 | 1 | 0 |
| 4 | 1 | 3 | 3 | 1 | 1 | 0 |
| 5 | 1 | 2 | 2 | 1 | 1 | 1 |
| 6 | 1 | 2 | 2 | 0 | 0 | 0 |
| 7 | 1 | 3 | 3 | 1 | 1 | 1 |
| 8 | 2 | 3 | 6 | 0 | 0 | 0 |
| 9 | 0 | 3 | 0 | 1 | 1 | 1 |
| 10 | 1 | 2 | 1 | 0 | 1 | 0 |
| 11 | 3 | 2 | 6 | 0 | 0 | 0 |
| 12 | 3 | 2 | 6 | 0 | 0 | 0 |
| 13 | 2 | 3 | 6 | 1 | 2 | 2 |
| 14 | 0 | 1 | 0 | 0 | 0 | 0 |
| 15 | 0 | 1 | 0 | 1 | 2 | 2 |
| 16 | 0 | 1 | 0 | 0 | 1 | 0 |
| 17 | 1 | 2 | 2 | 0 | 0 | 0 |
| 18 | 2 | 3 | 6 | 0 | 0 | 0 |
| 19 | 1 | 2 | 2 | 0 | 0 | 0 |
| 20 | 0 | 1 | 0 | 0 | 1 | 0 |
| 21 | 1 | 2 | 2 | 1 | 1 | 1 |
| 22 | 1 | 2 | 2 | 1 | 2 | 2 |

IHC score of HJURP in BCa and paracancerous samples

**Table S2:** RT-qPCR primer sequence

| HJURP Forward primer | GATTCAAAAAGCGGTGAGGTCG |
| --- | --- |
| HJURP Reverse primer | AGTCACACGTACATCCCTTCC |
| GAPDH Forward primer | GGAGCGAGATCCCTCCAAAAT |
| GAPDH Reverse primer | GGCTGTTGTCATACTTCTCATGG |

RT-qPCR primer sequence of HJURP and GAPDH

**Table S3:** shRNA Oligo Sequence

|  | Forward Oligo Sequence | Reverse Oligo Sequence |
| --- | --- | --- |
| shRNA1 | CCGGGCAAGTATGGAAGTTCGATATCTCGAGATATCGAACTTCCATACTTGCTTTTTG | AATTCAAAAAGCAAGTATGGAAGTTCGATATCTCGAGATATCGAACTTCCATACTTGC |
| shRNA2 | CCGGCAAAGTGACACCCTCGAAGTACTCGAGTACTTCGAGGGTGTCACTTTGTTTTTG | AATTCAAAAACAAAGTGACACCCTCGAAGTACTCGAGTACTTCGAGGGTGTCACTTTG |
| ShRNA3 | CCGGCCAAGAGCGATTCATCTTCATCTCGAGATGAAGATGAATCGCTCTTGGTTTTTG | AATTCAAAAACCAAGAGCGATTCATCTTCATCTCGAGATGAAGATGAATCGCTCTTGG |

Oligo Sequence of HJURP shRNA
